# Revision of the genus *Phloeoditica* Schedl - with description of two new genera and two new species in Phloeosinini (Coleoptera, Curculionidae, Scolytinae)

**DOI:** 10.3897/zookeys.56.522

**Published:** 2010-09-17

**Authors:** Bjarte H. Jordal

**Affiliations:** Natural History Collections, Bergen Museum, University of Bergen, NO-5007 Bergen, Norway

**Keywords:** Phloeoditica, Pseudoxylechinus, Asiophilus, Microditica, Phloeosinini, South-East Asia

## Abstract

The genus Phloeoditica Schedl currently includes four species from Southeast Asia. These species vary substantially in important morphological characters and indicate the existence of multiple genera for these species. A revision based on morphological and in part molecular data resulted in the transfer of Phloeoditica setosa to Pseudoxylechinus the erection of a new genus Asiophilus for Phloeoditica phloeosinoides and a new species Asiophilus macropunctatus from Vietnam. Another new genus with affinities to Phloeoditica is described based on the new species Microditica uniseriata from Thailand. The new genera are included in a revised key to the tribe Phloeosinini.

## Introduction

Schedl (1962, 1963) described the genus Phloeoditica based on the species Kissophagus curtus Eggers (1925). In the same publication he also transferred Kissophagus setosus Eggers (1939) to the same genus and described two new species, Phloeoditica elegans and Phloeoditica obscura. Browne (1966) added a fifth species, Phloeoditica phloeosinoides, which he with some hesitation placed in Phloeoditica. Phloeoditica obscura Schedl was later transferred to Pseudodiamerus by Wood (1988). The doubts expressed by Browne and the uncritical inclusion of Kissophagus setosus and Phloeoditica obscura in Phloeoditica by Schedl indicate a heterogeneous taxonomic history and a current uncertainty in the assembly of Phloeoditica species. Recent collections from Vietnam and Thailand have furthermore revealed two additional undescribed species with affiliations to some of the species currently included in the genus. A revision is therefore needed.

## Material and methods

Measurements follow the protocol by Wood (1982) and Jordal (1998). Four of the revised species were represented by only a single specimen in a condition which prevented genitalia and proventriculus preparations. Long series were available for the type species of Phloeoditica and one undescribed species in a closely related genus, which allowed dissections and DNA extraction. PCR amplification followed primers and protocols given in Jordal and Hewitt (2004).

Type material and other material are deposited in collections indicated by the following abbreviations:

CMNOCanadian Museum of Nature, Ottawa

NHCBNatural History Collections, Bergen Museum, Bergen

NHMWNatural History Museum, Vienna

NHRSNational Museum of Natural History (Riksmuseet), Stockholm

ZMUCZoological Museum, Copenhagen

## Results and discussion

### Phloeoditica

Schedl

Phloeoditica Schedl 1963: 260
Phloeoditica : Schedl 1962: 189 (Alonso-Zarazaga and Lyal 2009: unavailable name)

#### Type species:

Kissophagus curtus Eggers

#### Diagnosis.

Typical phloeosinine having a 5-segmented funicle and flattened club with oblique sutures and broadly separated pro- and mesocoxae. Readily recognised by a unique pair of long denticles at the outer apical margin of protibiae, and an unusually long and laterally curved inner uncus.

#### Description.

Size range 1.7–2.5 mm. Frons convex to moderately flattened in both sexes; antennal funicle 5-segmented, large club with two oblique sutures, the first marked by a septum, the second suture only marked by setae. Pronotum smooth, densely punctured, with short bristle-like setae. Scutellum not visible from above. Elytra with interstrial ground vestiture consisting of hair-like or scale-like setae; mesal grove immediately behind scutellum without interlocking nodules and cavities. Metepisternum clothed by plumose scales; sclerolepidia present, small and hair-like. Postnotum fused to metanotum; scutoscutellar suture parallel to scutellar groove for less than one-third of its length, then gradually curved laterally. Pleural suture nearly straight. Procoxae and metacoxae broadly separated. Outer apical angle of protibiae with a pair of projecting long socketed denticles, mostly embedded in cuticle; inner mucro extended into a large uncus curved towards outer margin.

#### Comments:

The name Phloeoditica is feminine as documented by Alonso-Zarazaga and Lyal (2009).

#### Taxa included:

Phloeoditica curta (Eggers, 1925), Phloeoditica elegans Schedl (1963).

#### Taxa excluded:

Phloeoditica phloeosinoides Browne (1966) to Asiophilus, Phloeoditica setosa (Eggers, 1939) to Pseudoxylechinus, Pseudoxylechinus obscura Schedl, 1963, to Pseudodiamerus (by Wood 1988).

### Phloeoditica curta

(Eggers, 1925)

Figs 1a 4a 5a 6a 7a

Kissophagus curtus Eggers 1925: 155
Phloeoditica curtus  (Eggers); Schedl 1962: 189

#### Type material examined.

##### Lectotype:

Burma with the following label data - “Kissophagus curtus n.sp. cotype, Eggers det 1924 / Mus. Pragense, Tenasserim, coll. Helfer / Cotype/ Dauerpräparat nr 2696 Fuehler, coll. Schedl” (NHMW) - current designation.

##### Other material examined:

Bangladesh, Khulna, Sunderbaans, Katka, ex Pongamia pinnata, 27 Feb. 1997, L. R. Kirkendall, leg (6 specimens, NHCB); Vietnam, with the following label: “Hoa-Binh (Tonkin) (A. de Cooman). Coll. J. Clermont” (1 specimen, NHMW). The locality in Hoa-Binh is located west of Hanoi in Vietnam, and not the island of Tonkin as incorrectly inferred by Wood and Bright (1992). Tonkin was the old colonial name of Vietnam.

**Figure 1.**
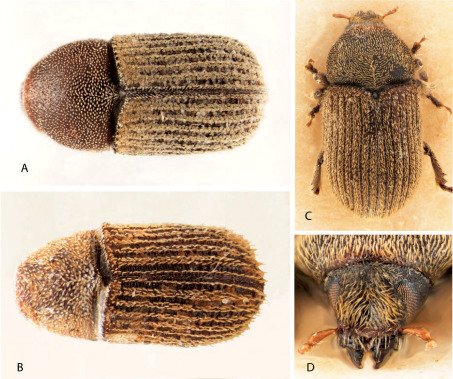
Dorsal and frontal view of Phloeoditica curta (**A**), Phloeoditica elegans (**B**), Pseudoxylechinus setosus (**C, D**).

#### Diagnosis:

Interstriae broad and strongly elevated, with about three irregular rows of rounded scales and one median row of longer bristles separated on average by their length. Apex of metatibiae truncated and slightly excavated, surrounded by 4–5 proximally pointing small spines and a larger inner mucro. It is readily distinguished from Phloeoditica elegans by the rounded scales on the interstriae. DNA sequences in Genbank: COI, GQ470889; EF1a, AF308402.

#### Description:

**Length** 2.1–2.5 mm, 1.9–2.1 times longer than wide. Colour dark brown, with yellow setae. **Head**. Frons slightly convex, transversely impressed above epistoma, a weaker and more narrow impressed area in central area, sometimes with a narrow and shallow median longitudinal groove towards upper level of eyes. Frons finely pubescent, setae coarse and slightly longer in impressed area, a few longer setae along epistomal margin. Eyes three times longer (dorso-ventrally) than wide, separated above by 2.2 times their width. Antennal club large, one oblique suture indicated by weak septum and a second false suture indicated by setae. Funiculus 5-segmented, scapus about three quarter length of funiculus and club combined. **Pronotum** 0.85 times as long as wide, constricted on anterior fourth, anterior margin and notum smooth, shiny, punctures separated by half their diameter; short bristle-like setae from each puncture. **Elytra** 1.4–1.5 times longer than wide, 1.9–2.0 times longer than pronotum, sides parallel on anterior two-thirds, apex subacuminate due to elevated interstriae 1 and 3. Base of elytra moderately procurved and elevated by a complete row of crenulations. Striae deeply impressed, punctures large, subquadrate, separated by half the length of a puncture and formed by transverse ridges. Interstriae as wide as striae, strongly elevated, interstriae 2 and 4 less elevated on declivity, punctures obscure, small granules of variable size mainly on declivity. Interstriae 10 not elevated, reaches level of metacoxae. Vestiture consisting of 2–3 irregular interstrial rows of densely placed rounded scales and one central row of longer bristles, each separated on average by their length. **Hind wing** with weakly pigmented veins, stigmal patch without apical tubercles or setae, six short setae along costal margin of stigma, cluster of five longer and softer costal setae at junction between R and SC-C. **Sclerolepidia** present along entire margin of metepisternum, small and hair-like; metepisternum with densely placed plumose scales. **Legs**. Procoxae separated by two-thirds the width of one coxa. Precoxal ridges very short, sharp. Mesocoxae separated by the width of one procoxa, mesocoxal process slightly proclinate. Metacoxae broadly separated. Protibiae armed by a long inner uncus curved towards outer margin and three socketed teeth embedded in cuticle along the outer lateral margin, the lower two close together and of similar size (type) or the inner tooth shorter (Bangladesh series). Mesotibiae armed by 4 lateral, socketed teeth. Metatibiae armed by 2 small lateral socketed teeth close to apex and 4–5 additional small apical spines along the outer and inner apical margin forming a semi-closed corbel-like structure. **Proventriculus** with apical plate about one-third as long as proventriculus, without median suture, with about 7 transverse and blunt ridges; femoral teeth small, irregularly placed; closing teeth few, long and soft; mastigatory brush rather weakly developed. **Aedeagus** about 5 times longer than wide, narrowly rounded at apex; apophyses about 2.5 times longer than aedeagal body (not clearly demarcated); a pair of short and narrow terminal plates present at apex; long inflated internal sac reaching half way between the apophyses, lightly sclerotised at apex. Tegmen a closed simple ring. Spiculum gastrale robust and L-shaped, with a tiny knob at posterior angle, about 0.8 as long as aedeagus. **Female rectum** with loop.

#### Comments.

The type locality in Tenasserim is most likely in the province Tanintharyi of Myanmar (Burma). A designation of a lectotype is necessary because syntypes are mixed with specimens from different localities. The series examined from Bangladesh differ marginally from the type series collected in Myanmar and Vietnam by the smaller inner tooth at the protibial apex.

### Phloeoditica elegans

Schedl, 1962

Figs 1b 4b 5b

Phloeoditica elegans Schedl 1962: 190

#### Type material examined.

2 paratypes: Vietnam with the following label data - “Tonkin, reg. de Hoa-Binh, A. de Cooman / Dauerpräparat nr 2081 Fuehler, coll. Schedl” and “Museum Paris, Tonkin, reg de Hoa-Binh, A. de Cooman 1929 / Dauerpräparat nr 2356 Fuehler, coll. Schedl” (NHMW).

#### Diagnosis:

Distinguished from Phloeoditica curta by the smaller size and the less elevated interstriae having a single row of erect hair-like bristles on interstria 1 and irregular rows of hair-like bristles on the remaining interstriae. The first suture of the antennal club has a more distinct septum visible without preparation.

#### Description:

**Length** 1.7–2.0 mm, 2.0–2.2 times longer than wide. Colour dark brown, with yellow setae. **Head**. Frons convex, transversely impressed on lower half, especially just above epistoma. Short bristle-like setae evenly distributed from upper level of eyes to epistoma. Eyes three times longer (dorso-ventrally) than wide, separated above by 2.2 times their width. Antennal club large, one oblique suture indicated by a distinct septum and a second false suture indicated by setae only. Funiculus 5-segmented, scapus about three quarter length of funiculus and club combined. **Pronotum** 0.9 times as long as wide, weakly constricted on anterior third, anterior margin and notum smooth, shiny, punctures deep, subconfluent; short spatula-shaped setae from each puncture. **Elytra** 1.5 times longer than wide, 1.9 times longer than pronotum, sides slightly diverging posteriorly, rounded behind. Base of elytra moderately procurved and elevated by a complete row of crenulations. Striae slightly impressed, punctures large, deep, transversely oval, separated by half the length of a puncture. Interstriae slightly wider than striae, slightly elevated, punctures at base of erect setae obscure. Interstriae 10 sharply elevated, reaches level of metacoxae. Vestiture consisting of 2–3 irregular interstrial rows of recumbent bristle-like setae slightly longer than distance between them and one central row of longer erect bristles each separated on average by 2–3 times their length. **Sclerolepidia** present along entire margin of metepisternum, small and hair-like; metepisternum with densely placed plumose scales. **Legs**. Procoxae separated by one-third the width of one coxa. Precoxal ridges very short, sharp. Mesocoxae separated by the width of one procoxa, mesocoxal process proclinate. Protibiae armed by a long inner uncus curved laterally and three socketed teeth embedded in cuticle along the outer lateral margin, the lower two of similar size and closer together, much larger. Mesotibiae armed by 4 lateral, socketed teeth. Metatibiae armed by 3 lateral socketed teeth close to apex.

#### Comments.

Types were mounted on cardboard which prevented examination of posterior ventral body parts. Types were not dissected for internal characters.

### Pseudoxylechinus setosus

(Eggers, 1939) comb. n.

Figs 1c, d 4c

Kissophagus setosus Eggers 1939: 3
Phloeoditica setosa  (Eggers); Schedl 1962: 188

#### Type material examined.

Holotype: Burma with the following label data - “N. E. Burma, Kambaiti, 7000ft., 1.5.1934, R. Malaise/ Typus [red paper]/ Kissophagus setosus n. sp. Type Eggers det 1938 / 301 65”(NHRS). Other material examined: 1 specimen with the same data as holotype, status of specimen not specified (NHMW).

#### Diagnosis.

Distinguished from all genera in Phloeosinini by the strongly concave frons and further from Phloeoditica and Microditica genus nov. by the visible large and dome shaped scutellum, and by the gradually rounded lateral margin of the protibia having socketed teeth. It is distinguished from other Pseudoxylechinus species except Pseudoxylechinus indicus Wood by the strongly concave frons and hair-like ground vestiture, and further from Pseudoxylechinus indicus by the more obtusely rounded lower lateral margin of the impressed frons, by the broader pronotum, and by the broader interstriae.

#### Description:

**Length** 2.7 mm, 2.1 times longer than wide. Colour dark brown with yellow setae. **Head**. Frons deeply concave between eyes from epistoma to upper level of eyes, upper half of impressed area reticulate with minute granules, shining below. Vestiture consisting of moderately long coarse setae over impressed area, slightly longer setae along lateral margin, sparse, minute setae above eyes. Eyes three times longer (dorso-ventrally) than wide, separated above by 3 times their width. Antennal club flattened, with one septate suture; funiculus possibly 6-segmented. **Pronotum** 0.8 times as long as wide, constricted on anterior third, anterior margin and notum lightly rugose, reticulate, punctures shallow, confluent; vestiture consisting of dense hair-like setae interspersed by a smaller number of longer and coarser bristles. **Elytra** 1.5 times longer than wide, 2.1 times longer than pronotum, sides subparallel on anterior two-thirds, rounded behind. Base of elytra procurved and elevated by a complete row of crenulations. Striae weakly impressed, punctures small, shallow, separated on average by less than their diameter. Interstriae three times wider than striae, weakly elevated, feebly granulated, punctures obscure. Interstria 10 not elevated, short, reaching beginning of metepisternum. Vestiture consisting of 4–5 irregular interstrial rows of hair-like setae (ground vestiture) and one central row of longer bristles each separated on average by their length. **Sclerolepidia** present along entire margin of metepisternum as small plumose scales; metepisternum with densely placed plumose scales. **Legs**. Procoxae separated by about half the width of one coxa (covered in glue). Precoxal ridges very short, sharp. Protibiae armed by six socketed teeth along the lateral and apical margin, the uppermost teeth half way and the remaining five close to the apical margin, inner uncus distinctly curved caudally. Mesotibiae armed by 4 lateral, socketed teeth. Metatibiae armed by 3 small lateral socketed teeth close to apex.

#### Comments.

The holotype has lost both antennae and only a badly preserved microscope slide of the non-type specimen was available, indicating most likely a 6-segmented funicle. However, the similarity in other characters to the species of Pseudoxylechinus Wood and Huang (1986) is striking and the species undoubtedly belongs here. Some authors have noted that Pseudoxylechinus may be a synonym of the recently resurrected genus Longulus Krivolutskaya, but the two genera remained separate in the absence of sufficient type material (Mandelshtam et al. 2007). Most species of Pseudoxylechinus are nevertheless distinguished from Longulus by having a distinctly impressed lower male frons, particularly so in Pseudoxylechinus indicus and Pseudoxylechinus setosus (see Wood 1986).

The distribution of the genus ranges from Japan in the east, via Yunnan and Tibet to Darjeeling in the west (Wood 1986). Consequently Pseudoxylechinus setosus fits well within this geographical pattern.

### Asiophilus

Jordal gen. n.

urn:lsid:zoobank.org:act:936D10AE-DBA2-4ABB-9021-442FB0904F76

#### Type species:

Phloeoditica phloeosinoides Browne, 1966, by current designation.

#### Diagnosis:

A typical phloeosinine genus with 5-segmented funicle, flattened club with two oblique sutures and widely separated pro- and mesocoxae. It is readily distinguished from Phloeosinus by the entire eye and less produced outer apical margin of the protibiae, and by the ascending venter.

#### Description:

Body length 1.5–1.65 mm. Frons convex; eyes entire, distance between eyes 2.7–2.9 their width; funicle 5-segmented, antennal club large and moderately flattened, with two oblique sutures. Pronotum and elytra roughly punctured, with a pair of medial closely set erect setae. Scutellum large, flat, slightly sunken with a small depression in elytra around scutellum; elytral base procurved, raised with a single complete row of crenulations. Metepisternal setae hair-like or bifid. All coxae widely separated; protibiae with 5–6 lateral and apical socketed teeth. Venter ascending to meet elytral apex.

#### Etymology.

From the Greek word philos (having affinity for) and Asia, referring to the type localities in Vietnam and Philippines.

#### Comments.

Browne (1966) placed Phloeoditica phloeosinoides in Phloeoditica with much hesitation and referred to several characters that deviate from Phloeoditica curta and Phloeoditica elegans, e.g. the larger and dorsally visible scutellum, the scant vestiture, and two real sutures in the antennal club. Species of this genus bear some superficial resemblance to the hylesinine genus Ficicis, but is readily distinguished by the 5-segmented funicle and the lack of pronotal asperities. Taxa included: Asiophilus phloeosinoides (Browne) and Asiophilus macropunctatus Jordal, sp. n.

### Asiophilus phloeosinoides

(Browne, 1966) comb. n.

Figs 2a–c 4d 5c

Asiophilus phloeosinoides Browne 1966: 243.

#### Type material examined.

Holotype: “Philippines, Tawi Tawi, Tarawakan, north of Batu Batu, 21. Oct 1961, Noona Dan Exp. 61–62 / Caught by mercury-light 18.30–00.30”. The holotype is pinned on a minuten-pin.

**Figure 2.**
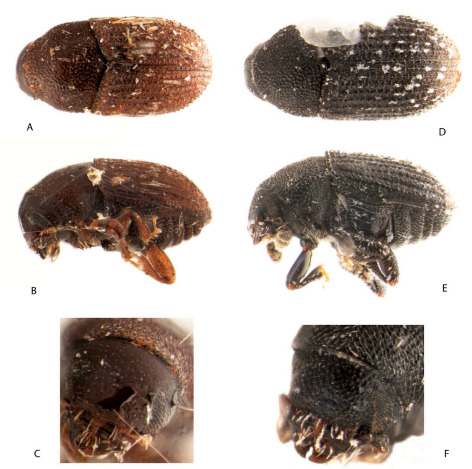
Dorsal, sinistral and front view of Asiophilus phloeosinoides (**A–C**) and Asiophilus macropunctatus (**D–F**).

#### Diagnosis.

Distinguished from Asiophilus macropunctatus by the more sparsely punctured frons, by the less asperate interstriae, by the scant elytral vestiture, and by the protibiae bearing 5 and not 6 lateral socketed teeth.

#### Description:

**Length** 1.5 mm, 2.0 times longer than wide. Colour dark reddish-brown. **Head**. Frons weakly convex above to flattened below, transversely impressed just above epistoma; punctures few, small and shallow. Vestiture consisting of 5–6 setae along epistomal margin, and almost invisible fine setae above. Eyes three times longer (dorso-ventrally) than wide, separated above by 2.7 times their width. Antennal club large, moderately flattened and oval, with two regularly spaced oblique sutures, finely pubescent. Funiculus 5-segmented, scapus about 0.8 times as long as the length of funiculus and club combined. **Pronotum** 0.85 times as long as wide, distinctly constricted on anterior third, notum roughly punctured, weakly granulated along anterior-lateral areas, punctures irregularly separated by about half their diameter; vestiture consisting of scant minute setae in punctures and a pair of medially placed longer erect setae. **Elytra** 1.3 times longer than wide, 1.7 times longer than pronotum, sides subparallel on anterior half, apex rounded. Base of elytra procurved and elevated by a complete row of crenulations. Striae impressed, punctures variably large, deep, subconfluent. Interstriae as wide as striae, slightly elevated, rugose, shining, with punctures about half the size of strial punctures and more widely spaced. Interstriae 10 reaching level of metacoxae. Vestiture consisting of interstrial rows of tiny recumbent setae. **Sclerolepidia** present along entire margin of metepisternum, very small; metepisternum almost glabrous, with scant minute setae. **Legs**. Procoxae separated by width of one coxa. Precoxal ridges short, distinct. Mesocoxae separated by slightly more than the width of procoxa, mesocoxal process vertical. Protibiae armed by 5 socketed teeth along the lateral and apical margin and one lightly curved inner mucro. Mesotibiae armed by 5 lateral, socketed teeth. Metatibiae armed by 4 lateral socketed teeth on apical half.

#### Comments.

This species is only known from the unique type specimen. The type locality Tawi Tawi is a small island just east of Sabah.

### Asiophilus macropunctatus

Jordal sp. n.

urn:lsid:zoobank.org:act:C7E42DF9-B578-4296-ABC1-4C0B1E293DA9

Figs 2d–f 4e 5d 

#### Type material.

Holotype: “Vietnam: Lao Cai, ca. 12km along road from Sapa to Lai Chau, 1950m, 22°20'58"N; 103°46'15"E, 1–12.V.1999, B. Hubley, pan traps (yellow), margin of 6m wide stream, edge of bamboo/2° forest”.

#### Diagnosis.

Distinguished from Asiophilus phloeosinoides by the more coarsely punctured frons and pronotum, by the elytral vestiture consisting of bristle-like interstrial setae, and by the protibiae bearing 6 lateral socketed teeth.

#### Description:

**Length** 1.65 mm, 2.0 times longer than wide. Colour dark brown or black. **Head**. Frons weakly convex, transversely impressed just above epistoma, punctures large, nearly confluent. Scant minute setae in punctures, about 12 longer setae along the epistomal margin. Eyes three times longer (dorso-ventrally) than wide, separated above by 2.9 times their width. Antennal club large, moderately flattened and oval, with two regularly spaced oblique sutures, finely pubescent. Funiculus 5-segmented, scapus about 0.8 times as long as the length of funiculus and club combined. **Pronotum** 0.8 times as long as wide, distinctly constricted on anterior third, notum roughly and densely punctured, punctures subconfluent; vestiture consisting of scant minute setae in punctures and a pair of median longer erect setae. **Elytra** 1.3 times longer than wide, 1.9 times longer than pronotum, sides subparallel on anterior two-thirds, apex rounded. Base of elytra strongly procurved and elevated by a complete row of crenulations. Striae impressed, punctures large, deep, separated by about one-quarter their diameter. Interstriae as wide as striae, slightly elevated, rugose, shining, with irregularly sized and more widely spaced punctures about half the size of strial punctures. Interstriae 8 and 9 on posterior half more elevated and sharply crenulated; interstriae 10 reaching level of metacoxae. Vestiture on disk consisting of interstrial rows of recumbent hair-like setae separated by less than their length, strial setae minute; on declivity interstrial setae in two confused rows. **Sclerolepidia** present along entire margin of metepisternum, very small; metepisternum with scant bifid setae, densely clothed closer to endosternum. **Legs**. Procoxae separated by width of one coxa. Precoxal ridges short, distinct. Mesocoxae separated by slightly more than the width of procoxa, mesocoxal process vertical. Protibiae armed by 6 socketed teeth along the lateral and apical margin and one lightly curved inner mucro. Mesotibiae armed by 6 lateral, socketed teeth. Metatibiae armed by 5 lateral socketed teeth on apical half.

#### Comments.

Only known by the unique type specimen from high altitude in Northern Vietnam.

### Microditica

Jordal gen. n.

urn:lsid:zoobank.org:act:284A0489-1042-4083-AB27-3BF7E41B399F

#### Type species:

Microditica uniseriata Jordal, sp. n., monotypic.

**Figure 3.**
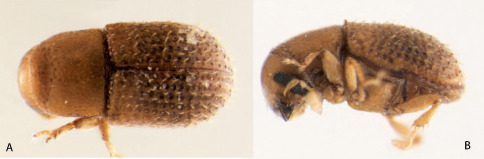
Dorsal and sinistral view of Microditica uniseriatus.

#### Diagnosis.

Typical phloeosinine genus with a 5-segmented funicle, barely visible scutellum and the broadly separated pro- and mesocoxae. The genus is diagnosed by the unique long and laterally curved inner uncus (mucro) of the protibiae, by the deeply grooved antennal club, and by the short crenulations at the elytral base reaching only to interstriae 5.

#### Description:

Frons convex and nearly glabrous in both sexes; eyes entire; funicle 5-segmented, antennal club large, with deeply grooved sutures. Pronotum smooth and shiny. Scutellum very small, flush with elytra and mainly visible on anterior slope. Elytral base nearly straight, with a single row of crenulations from scutellum to interstriae 5. Metepisternal setae scale-like; sclerolepidia distinct, small. Protibiae with the inner uncus very large and curved laterally, lateral teeth apparently unsocketed. Hind wings with four setae along costal margin at stigmal patch, hind margin from base to tip with long setae. All coxae widely separated, mesosternal process vertical. Postnotum fused to metanotum, scutoscutellar suture parallel to sutural groove for two-third of its length, then curves relatively abruptly laterally. Proventriculus simple, without apical sutural teeth or posterior mastigatory brush. Male aedeagus weakly sclerotised, spiculum gastrale weakly forked and tegmen a simple ring.

#### Etymology.

The Greek name micro (small) refers to the small size of a bark boring beetle; ditica is a Latinised form of the Greek feminine adjective dytiké (that likes to penetrate) (Alonso-Zarazaga and Lyal 2009).

#### Comments:

This genus shares most characteristics with Phloeoditica but is readily distinguished by the differently shaped protibia, the incomplete row of crenulations at the base of elytra, by the lack of ground vestiture, by the number and position of setae on the front and hind margin of the hind wings, and by the scutoscutellar suture following the scutellar grove much longer posteriad. The long setae along the hind margin are typical for small sized beetles (Kuschel et al. 2000) and may not be of significant phylogenetic value. Phylogenetic analyses of combined COI and EF-1α nucleotide data show that Microditica and Phloeoditica are quite unrelated and do not necessarily form a monophyletic group (unpublished data). This taxon share a few characters with other tribes such as Phloeotribini (semiarticulated antennal club) and Hypoborini (interrupted row of crenulations at base of elytra), but is readily distinguished from all taxa in those tribes by the broadly separated coxae.

**Figure 4.**
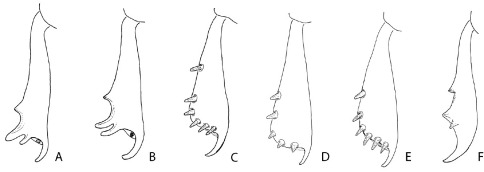
Posterior face of the left protibia of Phloeoditica curta (**A**), Phloeoditica elegans (**B**), Pseudoxylechinus setosus (**C**), Asiophilus phloeosinoides (**D**), Asiophilus macropunctatus (**E**), Microditica uniseriatus (**F**).

**Figure 5.**
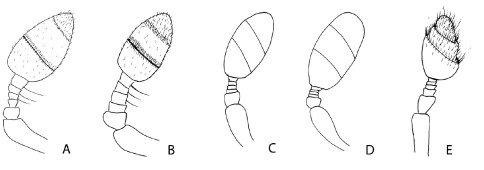
Antennal club and funicle ofPhloeoditica curta (**A**), Phloeoditica elegans (**B**), Asiophilus phloeosinoides (**C**), Asiophilus macropunctatus (**D**), Microditica uniseriatus (**E**).

**Figure 6.**
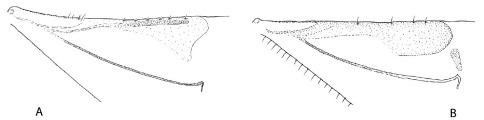
Basal part of hind wings in Phloeoditica curta (**A**)and Microditica uniseriata (**B**).

**Figure 7.**
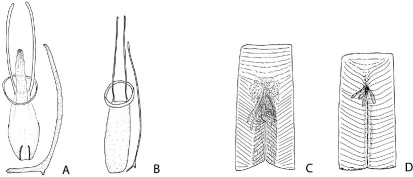
Male genitalia with spiculum gastrale (**A–B**) and proventriculus (**C–D**) in Phloeoditica curta (**A, C**) and Microditica uniseriata (**B, D**).

### Microditica uniseriata

Jordal sp. n.

urn:lsid:zoobank.org:act:F4626599-BD94-43CF-B48C-24B13035E8C3

Figs 3 4f 5e 6b 7b, d 

#### Type material.

Holotype: Thailand, Railay Beach - Krabi, 8.014N; 98.840E, 21 Nov. 1999 K. Harkestad, leg. (NHCB). Paratypes: Same locality as holotype (8 specimens, NHCB).

#### Diagnosis.

Very small body size; frons convex and shining in both sexes; large club with two deep grooves; protibiae with large inner uncus directed laterad; base of elytra with a single row of crenulations between scutellum and interstriae 5; uniseriate rows of interstrial spatulate bristles. DNA sequences in Genbank: COI, GQ470890; EF1a, GQ470891)

#### Description.

**Length** 0.85–0.95 mm, 2.0–2.1 times longer than wide. Colour light brown. **Head**. Frons convex, shiny, sparsely dotted by small shallow punctures separated by 2–4 times their diameter. Vestiture consisting of sparse minute hair-like setae, a few longer setae close to epistoma. Eyes 2.5 times longer (dorso-ventrally) than wide, separated above by 2.4 times their width. Antennal club large, two segments clearly marked by deep grooves, almost articulated; funiculus 5-segmented; scapus about 0.7 times as long as funiculus and club combined. **Pronotum** 0.95 times as long as wide, broadly rounded anteriorly, constriction on anterior fourth vaguely indicated. Disk smooth, shiny, with small and shallow punctures variably separated by 1–3 times their diameter. Vestiture consisting of scant minute hair-like setae and two median pairs and two anterior pairs of longer setae. **Elytra** 1.2 times longer than wide, 1.6 times longer than pronotum. Sides subparallel on anterior half, apex rounded. Base of elytra straight, with a single row of raised crenulations between scutellum and interstriae 5. Striae lightly impressed, punctures large, deep, separated by half their diameter. Interstriae smooth, punctures obscure, with a single row of small granules mainly on declivity and associated with the base of setae. Interstriae 10 not elevated, reaching level of metacoxae. Vestiture consisting of interstrial rows of erect spatulate bristles, each separated on average by their length. **Sclerolepidia** present along entire metepisternal suture, sparse metepisternal scale-like setae. **Legs**. Procoxae separated by width of 1 coxa; precoxal ridges very short but distinct. Mesocoxae 1.2 times wider than one procoxa; mesocoxal process vertical. Metacoxae broadly separated. Protibiae with two lateral unsocketed teeth, a third tooth just mesal to the second tooth, then gently curved to meet a large and laterally curved inner uncus. Mesotibiae and metatibiae each armed by 4 lateral socketed teeth. **Proventriculus** simple, apical plate short, about one-quarter of total length, with 3–4 transverse blunt ridges or rugae; femoral teeth weakly developed at base of 4–5 closing teeth. Mastigatory brushes not developed. **Aedeagus** weakly sclerotised, about 6–7 times longer than wide, apophyses very thin, longer than aedeagal body; tegmen a simple ring. Spiculum gastrale slightly shorter than aedeagus, weakly forked in the caudal end.

#### Etymology.

The specific epithet uniseriata refers to the uniseriate rows of interstrial bristles.

#### Comments.

Microditica uniseriata has been collected only from the type locality and was taken from the bark of an unknown dead shrub together with Hypothenemus birmanus (Eichhoff).

## Key to the genera of Phloeosinini of the World

**Table:** 

1.	Eye entire	2
–	Eye emarginate or divided	10
2.	Protibiae slender, armed by three apparently unsocketed teeth (denticles largely embedded in cuticle), inner uncus very large and long, curved towards outer margin (Figs 4a–b, f)	3
–	Protibiae broader, armed by at least four lateral socketed teeth of equal size, inner uncus/mucro shorter, directed caudally or nearly straight (Figs 4c–e)	4
3.	Protibiae armed on outer apical angle by two closely set projecting teeth and a smaller tooth on lateral margin (Figs 4a–b); crenulations at elytral bases reaching humeral angles; interstrial ground vestiture consisting of hair-like setae or scales (Figs 1a)	Phloeoditica
–	Protibia armed by two lateral teeth of equal size, and one mesal smaller tooth (Fig 4f); crenulations at elytral bases reaching interstriae 5; ground vestiture absent (Fig 3)	Microditica
4.	Funicle 5-segmented	5
–	Funicle 6- or 7-segmented	7
5.	Antennal club symmetrical (Figs 5c–d); crenulations at elytral base a single row (Fig. 2)	Asiophilus
–	Antennal club strongly asymmetrical; additional elytral crenulations close to scutellum	6
6.	Antennal club without sutures	Chramesus
–	Antennal club with two strongly procurved sutures	Pseudochramesus
7.	Funicle 7-segmented	8
–	Funicle 6-segmented	9
8.	Scutellum visible; base of elytra strongly procurved; procoxa broadly separated	Dendrosinus
–	Scutellum not visible, base of elytra weakly procurved, procoxae contiguous	Hyleops
9.	Body slender, about 2.1 times longer than wide; antennal club symmetrical	Carphotoreus
–	Body stout, about 1.6 times longer than wide; club strongly asymmetrical	Catenophorus
10.	Procoxa contiguous; antennal club subglobular; funicle 5- or 6-segmented	Cladoctonus
–	Procoxa separated; antennal club elongated; funicle 5-segmented	11
11.	Interstriae 10 extended to level of ventrite 3; humeral angles of elytra extended anteriorly, with largest crenulations	Phloeocranus
–	Interstriae 10 reaching level of metacoxae; elytral bases moderately procurved, with a single row of equally sized crenulations	12
12.	Pronotum finely asperate; antennal club with transverse sutures; eyes broadly emarginate	Phloeosinopsoides
–	Pronotum smooth; club with oblique sutures; eyes deeply emarginated or divided	13
13.	Eyes divided or nearly so; tarsal segment 3 slender	Hyledius
–	Eyes deeply emarginated; tarsal segment 3 broad and emarginate	Phloeosinus

## Supplementary Material

XML Treatment for Phloeoditica

XML Treatment for Phloeoditica curta

XML Treatment for Phloeoditica elegans

XML Treatment for Pseudoxylechinus setosus

XML Treatment for Asiophilus

XML Treatment for Asiophilus phloeosinoides

XML Treatment for Asiophilus macropunctatus

XML Treatment for Microditica

XML Treatment for Microditica uniseriata
